# Parathyroid gland detection using an intraoperative autofluorescence handheld imager – early feasibility study

**DOI:** 10.3389/fendo.2023.1190282

**Published:** 2023-07-24

**Authors:** Khalid Mohamed Ali, Samantha A. Wolfe, Nimesh V. Nagururu, Stefanie Seo, Sung-Min Han, Yoseph Kim, Eugene Oh, Dong-Yun Kim, Bo Ning, Seung Yup Lee, Richard Jaepyeong Cha, Ralph P. Tufano, Jonathon O. Russell

**Affiliations:** ^1^ Department of Otolaryngology-Head and Neck Surgery, Johns Hopkins School of Medicine, Baltimore, MD, United States; ^2^ i2KIE LLC, Gaithersburg, MD, United States; ^3^ Department of Research and Development, Optosurgical, LLC, Columbia, MD, United States; ^4^ Office of Biostatistics Research, National Heart, Lung, and Blood Institute, National Institutes of Health, Bethesda, MD, United States; ^5^ Sheikh Zayed Institute for Pediatric Surgical Innovation, Children’s National Hospital, Washington, DC, United States; ^6^ Department of Electrical and Computer Engineering, Kennesaw State University, Marietta, GA, United States; ^7^ Department of Pediatrics, George Washington University School of Medicine and Health Sciences, Washington, DC, United States; ^8^ Head and Neck Endocrine Surgery, Sarasota Memorial Health Care System, Sarasota, FL, United States

**Keywords:** parathyroid glands (PG), near infrared autofluorescence, hypocalcaemia, hypoparathryroidism, thyroid surgery, parathyroid autofluorescence (AF)

## Abstract

**Introduction:**

Parathyroid glands may be compromised during thyroid surgery which can lead to hypoparathyroidism and hypocalcemia. Identifying the parathyroid glands relies on the surgeon’s experience and the only way to confirm their presence was through tissue biopsy. Near infrared autofluorescence technology offers an opportunity for real-time, non-invasive identification of the parathyroid glands.

**Methods:**

We used a new research prototype (hANDY-I) developed by Optosurgical, LLC. It offers coaxial excitation light and a dual-Red Green Blue/Near Infrared sensor that guides anatomical landmarks and can aid in identification of parathyroid glands by showing a combined autofluorescence and colored image simultaneously.

**Results:**

We tested the imager during 23 thyroid surgery cases, where initial clinical feasibility data showed that out of 75 parathyroid glands inspected, 71 showed strong autofluorescence signal and were correctly identified (95% accuracy) by the imager.

**Conclusions:**

The hANDY-I prototype demonstrated promising results in this feasibility study by aiding in real-time visualization of the parathyroid glands. However, further testing by conducting randomized clinical trials with a bigger sample size is required to study the effect on levels of hypoparathyroidism and hypocalcemia.

## Introduction

Identifying parathyroid glands (PTG) during surgery to preserve healthy glands or remove diseased ones depends mostly on the surgeon’s experience and understanding of the anterior neck anatomy. Accidental damage to or removal of the parathyroid gland can lead to hypoparathyroidism and hypocalcemia (1, 2). A study by Puzzielo et al (3)found that the rate of hypoparathyroidism following thyroid surgery was 28.8% (757 patients out of 2,631); transient hypocalcemia occurred in 27.9%, and permanent hypocalcemia in 0.9%. In the past, different techniques such as dynamic optical contrast imaging, optical coherence tomography, and Raman spectroscopy have been employed but they have had limited utility because they were difficult to use and had a high cost (4). Unlike these technologies, intraoperative near-infrared autofluorescence (NIRAF) (5)detection technology for parathyroid gland identification appears to be gaining popularity. Recently a randomized clinical trial was conducted by Rebaudet et al (6), where they found that the rate of temporary postoperative hypocalcemia rate between the NIRAF group and the control group utilizing only surgeon identification was 9.1% and 21.7% respectively.

## Materials and methods

**Figure 1:**
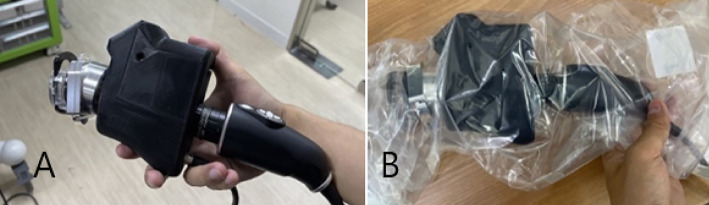
hANDY-I (Optosurgical, LLC) imaging system hand-held camera module: contains a coaxial, collimated 785 nm fiber laser module and a co-registered dual RGB/near-infrared (NIR) camera module. **(A)** Without sterile drape **(B)** Covered with sterile drape.

**Figure 2:**
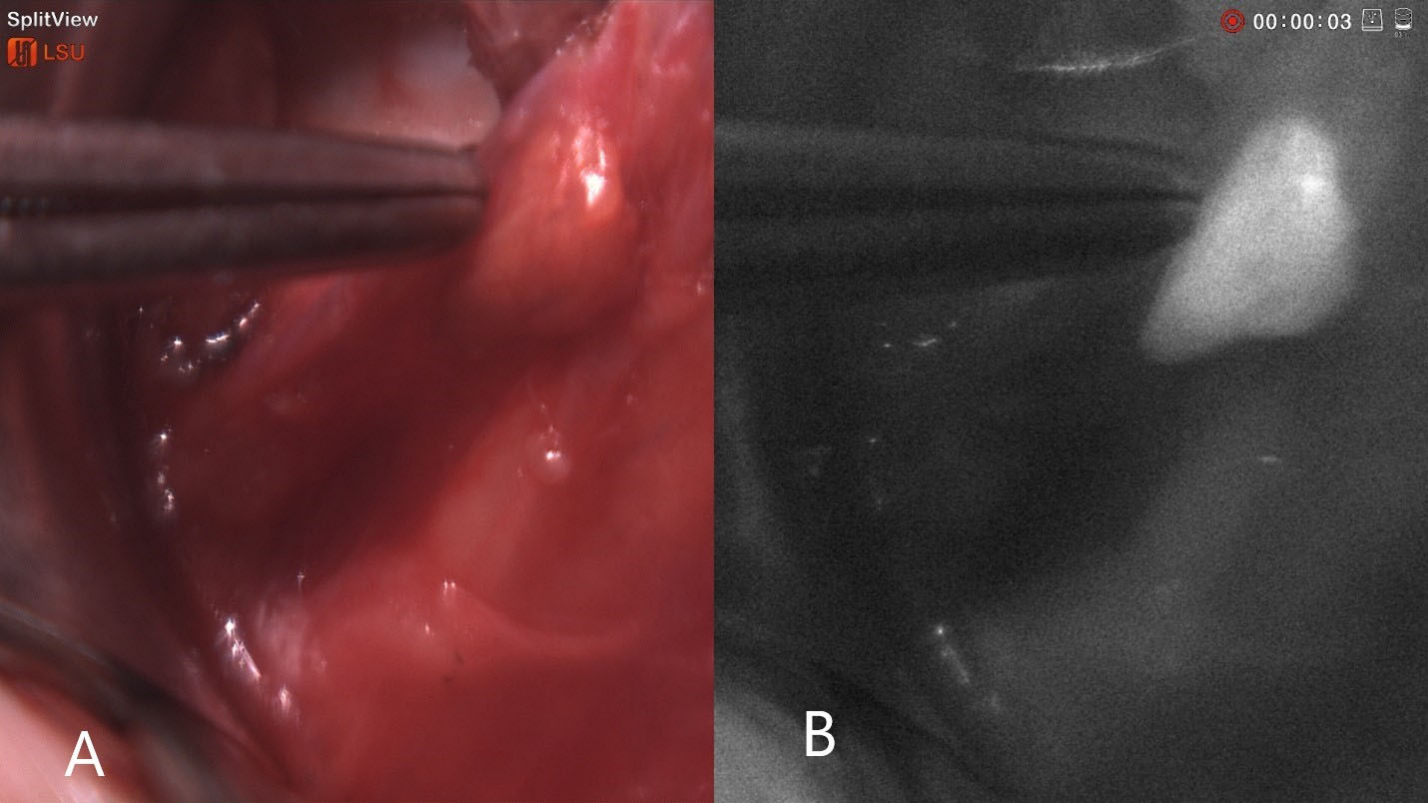
Intraoperative NIRAF of parathyroid glands: **(A)** Colored image of parathyroid glands **(B)** Autofluorescence image. The parathyroid tissue showing a well-recognizable homogenous autofluorescence signal compared to surrounding tissue.

**Figure 3:**
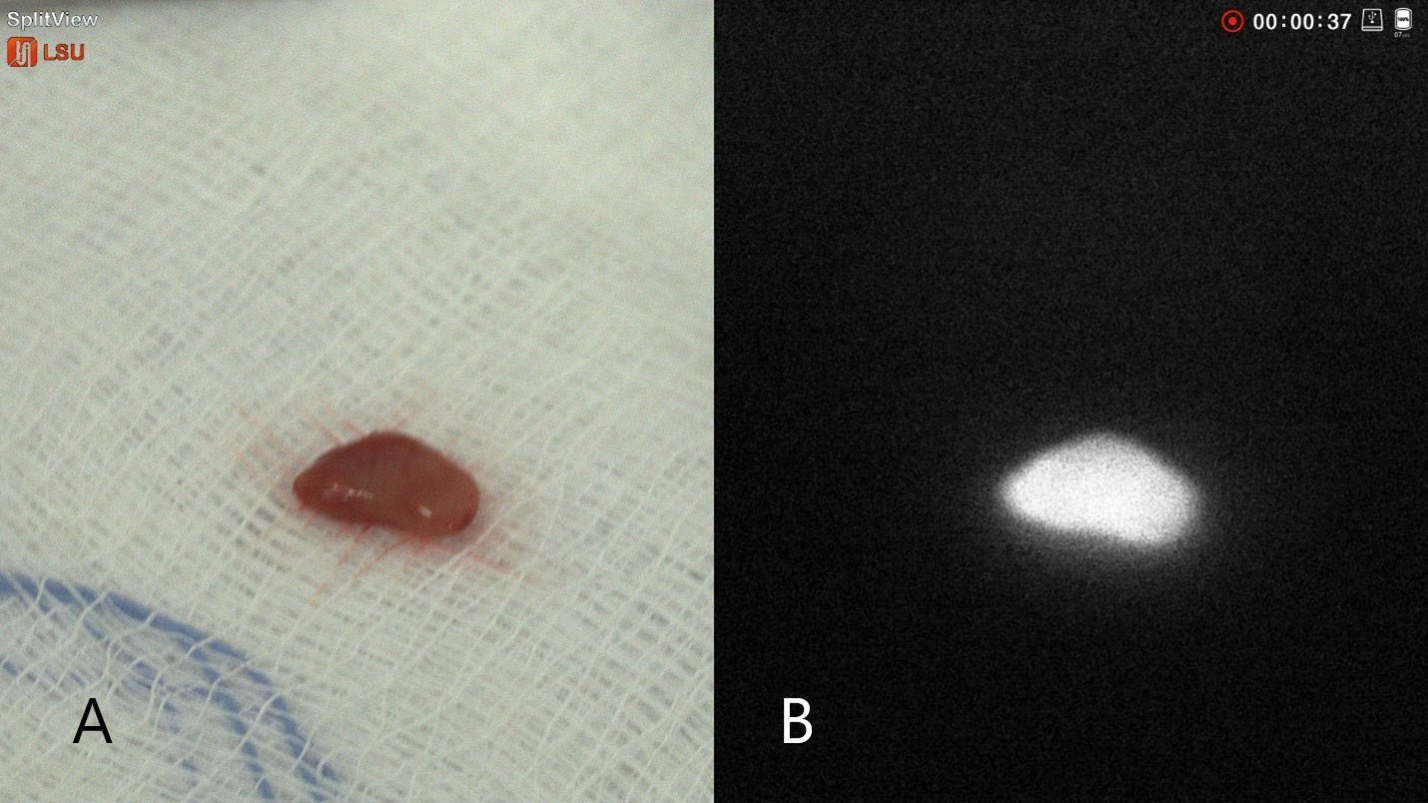
Intraoperative NIRAF of parathyroid glands *Ex-Vivo*: **(A)** Colored image of parathyroid glands **(B)** Autofluorescence image. The parathyroid gland showing clear homogenous autofluorescence signal.

We used a prototype NIRAF imager to prospectively collect data from 23 patients. The NIRAF imager named (hANDY-I) was developed and provided by Optosurgical, LLC. It consisted of a coaxial, collimated 785 nm fiber laser module and a co-registered dual red-green-blue (RGB)/near-infrared (NIR) camera module combined with a fixed c-mount lens (7) (**Figure 1**). The imager was used in order to obtain images and videos of the patients who underwent open thyroid surgery. camera was used *In-vivo* before and after tissue exposure and also *Ex-vivo* on resected samples as shown in (**Figures 2**, **3**).

In each surgery, potential parathyroid glands (PTGs) were examined by the surgeon with and without using the device. During PTG exploration it was recorded whether the tissue was discovered first with the aid of an imager or by naked eyes. The surgeon’s degree of confidence in PTG determination was self-assessed as high, medium, and low. The intensity of auto-fluorescence (high, low) and uniformity (homogeneous, non-homogeneous) were also recorded.

### Statistical analysis

Continuous variables were expressed as means and standard deviations (SD), and categorical variables as frequencies and percentages. McNemar test with continuity correction for paired proportions was used to compare the performance of surgeon and hANDY-I in identifying parathyroids during surgery. Welch’s two-sample t test was used to compare group means, and Fisher’s exact test was used to determine association between categorical variables. All statistical tests were two-sided. Statistical difference was deemed significant if the p value was smaller than 0.05. Due to hypothesis-generating nature of the study, no adjustment for type I error was made for multiple comparison. Statistical analyses were performed using the R software (R Foundation for statistical computing, version 4.0 and later).

## Results

Surgery data of 23 patients were included in this study involving 22 total thyroidectomies and 1 thyroid lobectomy. The average age of patients was 45 years (SD 16), and 91% (n=21) were women. The ethnicity was 70% White, 17% Black, and 13% Asian. The average BMI was 28 (SD 6) (**Table 1**). In all cases, a skilled, highly experienced otolaryngologist performed the surgery. A total of 75 PTGs were identified during the 23 cases. On average, 3.3 PTGs were identified per surgery. 98% of the PTGs that showed strong auto-fluorescence were correctly detected by hANDY-I. Overall, hANDY-I confirmed 71 out of 75 PTGs (95%) that were identified. In comparison, the surgeon identified 92% of the glands that the hANDY-I visualized. Thus, six glands (8%) that the hANDY-I identified had not been initially seen by the surgeon and would have been otherwise not identified.

**Table 1:** Baseline characteristics of patients.

	Study population (n=23)
Age in years (SD)	45.4 (15.9)
Women (%)	21 (91.3)
Body mass index (SD)	27.5 (6.3)
Ethnicity (%)	
Caucasian African American Asian	16 (69.6) 4 (17.4) 3 (13.0)
Surgery type (%)	
TTX LOB	22 (95.7) 1 (4.3)

TTX, Total thyroidectomy; LOB, Thyroid lobectomy.

We also compared the performance of hANDY-I and surgeon using the McNemar test (8) with continuity correction for paired proportions. We assumed that each of the 75 PTGs was an independent observation, regardless of patient or surgery type. The test shows that there is no statistically significant difference between hANDY-I and a skilled surgeon in identifying PTG during the surgery (p=0.75). This is consistent with the comparable marginal success rates of correct PTG identification by hANDY-I and the surgeon. The Jaccard similarity index is 0.88, once again supporting a high degree of agreement between the device and the surgeon regarding PTG identification (9).

**Figure 4:**
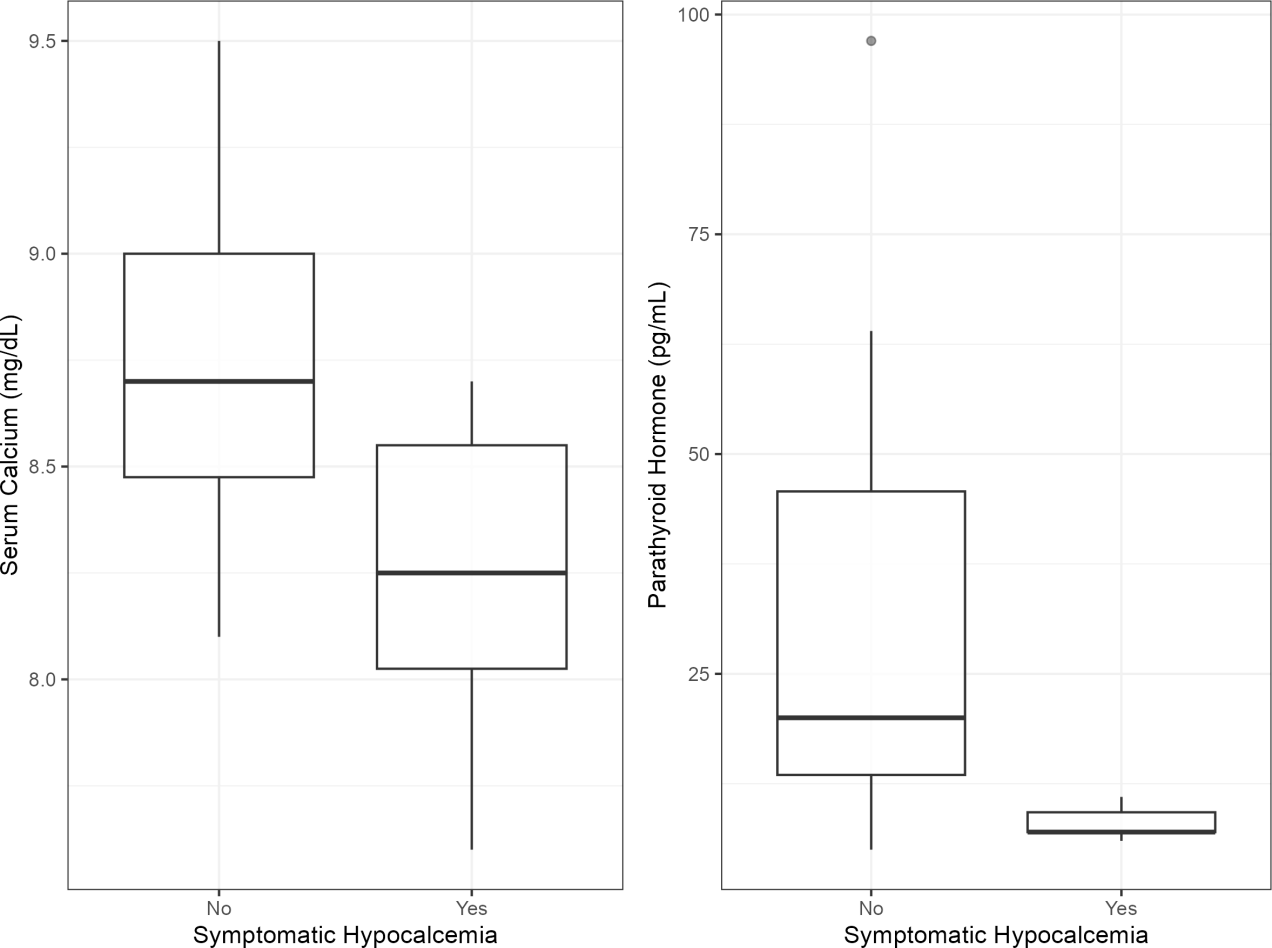
Side-by-side boxplots illustrating the relationship between [serum calcium/PTH] levels and symptomatic hypocalcemia status. Median (bold line), Interquartile Range (IQR) (box), and outlier (circle) are shown.

We reviewed the serum calcium and PTH levels of 22 patients who had at least one measurement. *After surgery, six patients (27%) showed symptoms associated with hypocalcemia while 16 patients (73%) did not.* The patients were divided into two groups: those who had post-op symptomatic hypocalcemia and those who did not. The average serum calcium and PTH levels of the two groups before and after surgery were summarized in **Table 2**. There was no statistically significant difference between the two groups in pre-op serum calcium (p=0.56) and PTH levels (p=0.51). On the other hand, there was a significant difference in both post-op serum calcium (p=0.03) and PTH levels (p<0.01) between symptomatic and asymptomatic groups (**Figure 4**), which is expected but does not corelate directly to the use of NIR. There was no significant association between symptomatic hypocalcemia and the surgeon’s confidence level (p= 0.27), the number of PTGs identified (p=0.54), or the number of tissues with auto-fluorescence (p=0.31).

**Table 2:** Serum calcium and PTH before and after surgery.

	Pre/Post Operation	n	Overall	Symptomatic Hypocalcemia		P value*
			Average (SD)	No (n=16)	Yes (n=6)	
Serum calcium	Pre Post	22 22	9.3 (0.4) 8.6 (0.4)	9.3 (0.3) 8.7 (0.4)	9.2 (0.5) 8.2 (0.4)	0.56 0.03
PTH	Pre Post	21 22	47.3 (18.8) 24.4 (23.4)	48.7 (20.0) 30.6 (24.8)	42.8 (15.5) 8 (2)	0.51 <0.01

*Welch’s two-sample t-test; comparison between symptomatic and non-symptomatic groups.

## Discussion

Hypoparathyroidism and hypocalcemia are still the most common complications following thyroid surgery, affecting up to 30% of patients (10, 11). This may be due to the parathyroid glands’ small size and numerous variations in their location. This opens the door to introduce new tools that can aid surgeons in identifying these glands, in order to decrease the complication rate and the need for additional treatment which may burden patients physically and financially.

NIRAF technology may be useful in this regard as it offers a non-invasive real-time tool to help in the identification of the parathyroid glands. The technology was described in 2008 (12)and tested *in vivo* in 2013 by McWade et al. (13, 14). While the source of the fluorophores is not completely understood, McWade hypothesized initially that it could originate from calcium-sensing receptors due to their high concentration in the parathyroid glands, this was later debunked. Since then, multiple scholars (**Table 3**) have studied this novel technology.

**Table 3:** Review of some of the previous studies that utilized optical near infrared autofluorescence for parathyroid glands detection.

Author	Institute/Country	Year published	Number of Participants	Detection percentage	Sensitivity	Notes
McWade (14)	Vanderbilt University/USA	2014	110	100%	100%	First study to report on the use of optical NIRAF imaging system.
Falco (15)	University of Buenos Aires/Argentina	2016	28	100%	100%	Used FDA approved device Fluobeam 800 (Fluoptics).
De Leeuw (16)	Université Paris-Saclay/France	2016	35	98%	99%	Used Fluobeam 800 (Fluoptics), reported higher AF signal *in vivo* than ex vivo
Kim (17)	Kosin University/South Korea	2016	8	100%	N/A	Lab-built system was used.
Ladurner (18)	Ludwig-Maximilians-Universität München/Germany	2017	25	81%	100%	Lab built using Karl Storz NIR/ICG camera
Shinden (19)	Kagoshima University/Japan	2017	17	100%	100%	Used new optical system PDE (Hamamatsu)
Kahramangil (20)	Cleveland clinic/USA	2017	44	98%	N/A	Used Fluobeam 800 and ICG fluorescence to evaluate PGs perfusion.
Benmiloud (21)	Hôpital Européen de Marseille/France	2018	93	76%	N/A	Used Fluobeam 800 (Fluoptics), focused on postoperative calcium and PTH levels.
Kahramangil (22)	Multicenter	2018	210	98%	N/A	Multicenter study using Fluobeam.
Kose (23)	Cleveland clinic/USA	2019	50	96%	96.5%	Used Fluobeam 800 (Fluoptics) to examine 199 PGs.
Squires (24)	The Ohio state university/USA	2019	59	87%	87%	Used new precommercial device PDE Neo II (Hamamatsu).
DiMarco (25)	Hammersmith Hospital/UK	2019	96	90.5%	98%	Used Fluobeam 800 (Fluoptics) and targeted parathyroidectomy cases.
Kim (7)	Johns Hopkins Hospital/USA	2019	26	98%	N/A	Used hANDY-I (Optosurgical).
Kose (26)	Cleveland clinic/USA	2020	310	98%	98.5%	Used Fluobeam 800 (Fluoptics), found that 21% of abnormal glands were recognized with NIRAF first.
Papavramidis (27)	Aristotle University of Thessaloniki/Greece	2021	180	N/A	N/A	Randomized-controlled trial used Fluobeam LX (Fluoptics) found reduced rates of unintentional PG excision with NIRAF used.

NIRAF, Near Infrared Autofluorescence; FDA, Food and Drug Administration; ICG, Indocyanine green; PG, Parathyroid gland; PTH, Parathyroid hormone; N/A, Not applicable or not reported.

While some researchers have found a decrease in the rate of temporary postoperative hypocalcemia rate from 22% to 9% and decreased need for parathyroid autotransplantation rates from 16% to 4% (6), this was not always the case as reported in a study by DiMarco et al (28), who found no difference in the rates of postoperative hypocalcemia between NIRAF and non-NIRAF groups. In some cases, tissue exposure was not needed as the parathyroid AF signal was picked up before the gland itself was clearly visible to the naked eye. This is due to the increased tissue penetration, low tissue damage, and increased focus of NIR (22, 29). The aim of this technology is to help in decreasing the rates of temporary and permanent hypocalcemia and hypoparathyroidism and decrease the need for confirmatory studies.

Currently there are two NIRAF parathyroid detection devices approved by the FDA and commercially available. The first one is the Fluobeam LX from Fluoptics, which is an image-based device. It has a sensitivity ranging from 91% - 98% (16, 22, 23). One limitation of Fluobeam is that it is sensitive to light necessitating that all light sources be turned away from the field, which can disrupt the surgery and add time to the procedure. The second device is PTeye from Medtronic, which is a probe-based device. This device has a similar accuracy (94%) (30). It utilizes a small probe that can fit through small incisions because it requires direct contact with the tissue. It provides a quantified parathyroid fluorescence but requires calibration for each patient which can affect the results if not done correctly. PTeye also lacks video output, which means that the surgeon may spend more time trying to locate the glands than confirming them with the probe.

While most experienced high-volume thyroid surgeons are comfortable operating without such devices, the technology could benefit low-volume surgeons who may be less familiar with anatomic variations in parathyroid location. NIRAF technology could become a standard practice by surgeons if it is proven to add value and decrease the rate of parathyroid gland injury. This is similar to how intraoperative nerve monitoring is now being utilized by 83–93 percent of American head and neck surgeons and international endocrine surgeons (31), despite their knowledge of recurrent laryngeal nerve anatomy.

While the technology is promising, NIRAF has some limitations. For example, false positives and false negatives can occur, potentially leading to the removal of healthy tissue or incomplete removal of diseased glands. Moreover, the depth of penetration could be a limiting factor that could result in an incomplete visualization and assessment of the parathyroid glands depending on how much of it is exposed. Han et al. reported that the depth of penetration of NIARF could reach a maximum of 3 mm in unexposed tissue with an average of 1.03 mm (32). Another limitation is the variability of the fluorescence intensity and homogeneity of PGs that could result from the underlying pathology, tissue exposure or other factors like BMI and preoperative Ca and Vitamin D levels (33). Lastly, the viability of the parathyroid gland cannot be assessed by autofluorescence imaging.

In this study, our aims were to evaluate the feasibility of the prototype, the learning curve of its use in the hands of a high-volume surgeon and to collect as much data as possible from our first 23 cases. We have successfully demonstrated the usefulness of the new device in identification of parathyroid glands *in situ* by offering color and autofluorescence images in real time during the surgery. We also showed that the overall accuracy is comparable to that of a highly skilled surgeon, and the data suggests that the accuracy could be even higher for parathyroid glands that show strong autofluorescence signal. To establish statistical equivalence (or even superiority) of the device compared to surgeons, however it may require an elaborate clinical trial with larger sample size and participation of multiple surgeons. It is beyond the scope of the study.

The current prototype is a hand-held imager that has a 2-sensor camera with an attachable sterile drape adapter that can be easily attached by the scrub technologist or surgical assistant. The camera module is connected to a monitor that shows both colored and autofluorescence images side by side which can help the surgeon in knowing exactly where the camera is pointed **Figures 2**, **3**. The device does not require tissue contact in order to assist in localizing the parathyroid glands and it is not affected by the operating lights. We were able to obtain autofluorescence signals before dissecting the parathyroid gland even when it was partially covered by fat and/or fascia.

**Figure 5:**
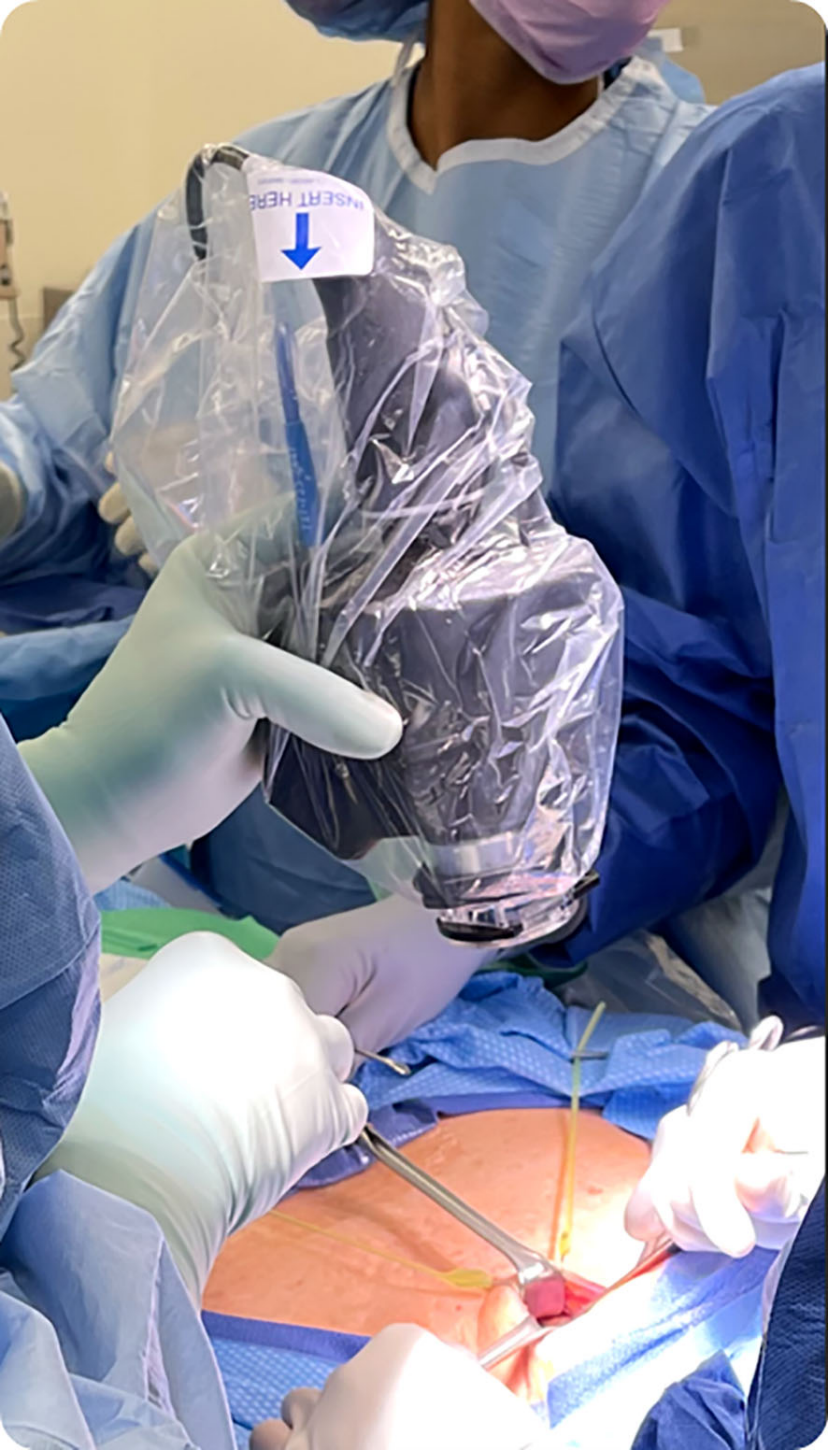
NIRAF imager covered by sterile drape and being used by the surgeon intraoperatively.

There is a slight learning curve to using the hand-held device. For example, the surgeon should orient the probe towards the head of the bed and position the device around 6 inches above the surgical field. It has been noted by surgeons that the current prototype feels heavy or bulky for extended handling and use (**Figure 5**). This shortcoming of the prototype will be fully addressed in the final product.

There are a few limitations in our study. First, the study is based on a small sample size. This limitation will be resolved by ongoing intraoperative utilization, and we plan to publish findings with an increased sample size in the future. Another limitation was the difficulty in independently determining the success rates of naked eye identification and the imager. This is due to time constraints in the operating room and the lack of an independent medical staff for imager-only identification. Pathological confirmation was not possible in all the cases as it is not a standard practice and could lead to damage or devascularization of healthy PTGs.

## Conclusion

NIRAF technology is a useful addition to thyroid surgeons’ arsenal that may help decrease the rates of accidental damage or removal of the parathyroid glands. The hANDY-I prototype shows promising results in this early feasibility study in terms of visualizing the parathyroid glands, and we aim to continue to test it in order to accurately assess its specificity and sensitivity. Further evaluation by conducting randomized clinical trials is needed to validate the usefulness of this technology and how it can affect the rates of hypocalcemia and hypoparathyroidism.

## Data availability statement

The raw data supporting the conclusions of this article will be made available by the authors, without undue reservation.

## Ethics statement

The studies involving human participants were reviewed and approved by Johns Hopkins IRB. Written informed consent for participation was not required for this study in accordance with the national legislation and the institutional requirements.

## Author contributions

KA, EO, RC, SL, RP and JR contributed to conception and design of the study. SH and DK contributed to the design of the study. KA and YK performed data acquisition. SH performed the statistical analysis. KA and SW wrote the first draft of the manuscript. SS, SH, NN and BN wrote sections of the manuscript. All authors contributed to the article and approved the submitted version.
